# Soy isoflavones, estrogen therapy, and breast cancer risk: analysis and commentary

**DOI:** 10.1186/1475-2891-7-17

**Published:** 2008-06-03

**Authors:** Mark J Messina, Charles E Wood

**Affiliations:** 1 Nutrition Matters, Inc, 439 Calhoun Street, Port Townsend, WA 98368, USA; 2 Department of Pathology/Section on Comparative Medicine, Wake Forest University, School of Medicine, Winston-Salem, NC, USA

## Abstract

There has been considerable investigation of the potential for soyfoods to reduce risk of cancer, and in particular cancer of the breast. Most interest in this relationship is because soyfoods are essentially a unique dietary source of isoflavones, compounds which bind to estrogen receptors and exhibit weak estrogen-like effects under certain experimental conditions. In recent years the relationship between soyfoods and breast cancer has become controversial because of concerns – based mostly on in vitro and rodent data – that isoflavones may stimulate the growth of existing estrogen-sensitive breast tumors. This controversy carries considerable public health significance because of the increasing popularity of soyfoods and the commercial availability of isoflavone supplements. In this analysis and commentary we attempt to outline current concerns regarding the estrogen-like effects of isoflavones in the breast focusing primarily on the clinical trial data and place these concerns in the context of recent evidence regarding estrogen therapy use in postmenopausal women. Overall, there is little clinical evidence to suggest that isoflavones will increase breast cancer risk in healthy women or worsen the prognosis of breast cancer patients. Although relatively limited research has been conducted, and the clinical trials often involved small numbers of subjects, there is no evidence that isoflavone intake increases breast tissue density in pre- or postmenopausal women or increases breast cell proliferation in postmenopausal women with or without a history of breast cancer. The epidemiologic data are generally consistent with the clinical data, showing no indication of increased risk. Furthermore, these clinical and epidemiologic data are consistent with what appears to be a low overall breast cancer risk associated with pharmacologic unopposed estrogen exposure in postmenopausal women. While more research is required to definitively allay concerns, the existing data should provide some degree of assurance that isoflavone exposure at levels consistent with historical Asian soyfood intake does not result in adverse stimulatory effects on breast tissue.

## Background

In 1990, participants of a workshop sponsored by the U.S. National Cancer Institute concluded that soybeans contain several putative chemopreventive agents [1]. In the years since, there has been considerable investigation of the potential for soyfoods to reduce risk of cancer, and in particular cancer of the breast. The basis for the initial focus on breast cancer can be attributed to several things: the historically low breast cancer incidence rates in Asia, where soyfoods comprise an important dietary component [2]; research demonstrating the potential for isoflavones – one of the putative chemopreventive agents identified in soybeans – to exert antiestrogenic effects [3]; early epidemiologic data showing an inverse association between soy intake and breast cancer risk [4]; and rodent studies showing a protective effect of soy intake against carcinogen-induced mammary cancer [5].

In recent years, however, the relationship between soyfoods and breast cancer has become controversial because of concerns that soy-derived isoflavones, which exhibit estrogen-like properties under certain experimental conditions, may stimulate the growth of existing estrogen-sensitive breast tumors [6]. These concerns exist because of evidence showing that isoflavones bind and transactivate estrogen receptors (ERs) [7,8], induce proliferation and estrogenic markers in MCF-7 cells, an ER positive (ER+) breast cancer cell line [9-14], and elicit estrogenic effects in rodent reproductive tissues [15,16]. In contrast to these findings, epidemiologic evidence shows that among Asian women, higher soy intake is associated with a nearly one-third reduction in breast cancer risk [17] and that Japanese breast cancer patients, in comparison to Western women, exhibit better survival rates even after controlling for stage of diagnosis [18-22].

In 2006, the American Cancer Society concluded that breast cancer patients can safely consume up to three servings of traditional soyfoods per day, although the group advised against the use of more concentrated sources of isoflavones such as powders and supplements [23]. Other expert views are less supportive of the use of any isoflavone-containing products for breast cancer survivors and in some cases for women at high risk of this disease [24-28]. Many women are understandably confused about whether to incorporate soy into their diet. Thus, there is a need for health professionals to have a better understanding of the current evidence relating to soy and breast cancer so that they can better advise their patients and clients. In this analysis and commentary we attempt to outline current concerns regarding estrogen-like effects of isoflavones in the breast and place these concerns in context of recent evidence regarding estrogen therapy (ET) use in postmenopausal women.

### Background on isoflavones

The three soybean isoflavones are genistein, daidzein, and glycitein. These non-steroidal compounds are naturally present in the soybean and non-fermented soyfoods primarily in their beta glycoside forms: genistin, daidzin, and glycitin. Throughout this paper isoflavone amounts refer to the aglycone weight, which is ~60% of the glycoside. In the soybean itself and in most soy products, genistin/genistein, daidzin/daidzein, and glycitin/glycitein account for approximately 50–55%, 40–45%, and 5–10% of total isoflavone content, respectively [29]. Older adults in Japan and Shanghai, China, typically consume between 25 and 50 mg/d isoflavones and probably no more than 5% of these populations consume ≥ 100 mg/d [30]. In contrast, people in the United States and Europe consume on average < 3 mg/d of isoflavones [31-33].

Isoflavones are diphenolic compounds with a chemical structure similar to estrogen that bind to both estrogen receptors alpha (ERα) and beta (ERβ) and, for this reason, are commonly referred to as phytoestrogens [34,35]. Isoflavones exhibit estrogen-like properties but bind more weakly to ERs than 17β-estradiol (E2), which is the primary physiologic estrogen. Genistein, which is the main circulating and best-studied isoflavone, transactivates ERα and induces estrogenic effects with ~10^3^-10^4 ^less potency than E2 [7,8]. However, serum isoflavone concentrations after a high-soy meal can reach low micromolar levels [36,37], thereby exceeding postmenopausal total estrogen concentrations by ~10^3 ^[38]. This evidence has contributed to the idea that isoflavones may potentially elicit estrogen-like effects and thus serve as a natural alternative to ET in postmenopausal women. Isoflavones also preferentially bind to and transactivate ERβ in comparison to ERα [9,39,40] and induce distinct changes in ER conformation [41], leading to speculation that they may function as selective estrogen receptor modulators (SERMs) [42-44]. Despite this designation, unlike different forms of estrogen [45-55], there is scant evidence for any clear estrogen-like or antiestrogenic-like effects of soyfood or isoflavone intake on the human breast or a number of other parameters [44,55-64].

### Effects of isoflavones on mammary/breast cell proliferation

#### Animal studies

Concern over the possible tumor-stimulatory effects of isoflavones is based largely on the proliferative effect of genistein on MCF-7 cells *in vitro *and in studies of mammary cancer in rodents. A variety of studies have shown that isoflavones stimulate ER+ human breast cancer cell xenoplants in ovariectomized athymic mice [13,65-68], estrogen-dependent mammary tumors in rats [69], and reproductive tissues in adult female mice [70,71]. Other research using rodent models has also demonstrated that genistein is the primary isoflavone responsible for tumor stimulation [72]; that more processed soy products result in faster tumor growth than less processed soy products [68]; and that genistein inhibits the efficacy of tamoxifen, a SERM used in the treatment and prevention of breast cancer [73].

Even in rodent models, however, isoflavones are generally weak estrogen agonists relative to E2. Most rodent studies use scaled doses at least 5 times the amount found in traditional Asian diets [30], and many studies have used direct injection of purified isoflavones, which results in substantially higher levels of unconjugated isoflavones than dietary administration [70]. Importantly, the isoflavone dose required for estrogen-like effects in women has yet to be identified despite three decades of study. So although isoflavones clearly act as estrogens in rodent models, relevant dose effects for human consumption are still very unclear.

There are several noteworthy limitations/weaknesses of the ovariectomized athymic mouse models used in many of the experiments noted above. First, the lack of immune function, which is a necessary element of these models, may eliminate a potential mechanism by which genistein reduces tumor development. Recent research in B6C3F1 mice shows that enhanced immune function resulting from pretreatment with genistein (20 ppm) is correlated with protection against chemically-induced mammary tumors [74]. Second, unlike postmenopausal women, ovariectomized mice do not produce sufficient endogenous estrogen to promote development and growth of estrogen-dependent tumors. Thus, the effects of isoflavones are occurring in an estrogen-depleted environment that does not accurately reflect conditions in either premenopausal or postmenopausal women. It has been argued that estrogenic and tumor-stimulatory effects of isoflavones may be evident only in this type of hypoestrogenic environment. However, this criticism has been addressed by two different models in which isoflavones still lead to tumor stimulation. In one, mice are implanted with MCF-7C_a _cells transfected with the enzyme aromatase, enabling the cells to synthesize estrogen; in the other model, mice are continually given small amounts of estrogen [75].

A third critique relates to isoflavone dose. In many studies showing estrogenic effects, mice are exposed to an amount of genistein (750 ppm) that greatly exceeds typical dietary intake. In Japan for example, adults consume about 15–20 mg genistein daily (total mean isoflavone intake is approximately 40 mg), which equates to a dietary concentration of about 30–40 ppm. When expressed on a caloric basis to adjust for differences in metabolism, the difference between human and rodent isoflavone exposure is ~8–16 times higher than the 25 – 50 mg per 1800 Kcal in a traditional Asian diet. (A 30 gm mouse consuming 3 gm of food/d with 750 ppm genistein will consume ~2.25 mg/d of isoflavones, which equates to ~405 mg per 1800 Kcal.) Exposure to purified genistein levels as low as 150 ppm has also been shown to stimulate MCF-7 cell growth, albeit to a lesser extent than higher genistein doses or E2 treatment [67]. Fourth, it is not clear to what extent the existing MCF-7 xenoplants in nude mice reflect tumors in breast cancer patients. These tumors are fully transformed and composed of cells that are extremely sensitive to the growth-stimulating effects of estrogen. Finally, other potentially relevant rodent models [76-78] have shown that isoflavones or isolated soy protein (ISP, by definition is >90% protein) suppress, rather than stimulate, the growth of tumors in mice implanted with MCF-7 cells and even enhance the efficacy of tamoxifen [79,80].

#### Clinical studies

Breast tissue is highly regulated by sex hormones, particularly estrogens and progestogens, and breast epithelial proliferation is widely used as an indicator of hormonal exposure or effect. Epithelial cell proliferation also serves as an important prognostic marker in human breast cancer [81] and may help predict risk associated with different hormonal agents [82]. A common method for evaluating proliferation is the immunohistochemical marker Ki67 (also called MIB1), which is a nuclear protein expressed by cells in all active phases of the cycle but not in quiescent or resting cells [83]. Ki67 labeling correlates significantly with higher carcinoma grade, clinical response to endocrine therapy, higher risk of relapse, and worse survival in patients with early breast cancer [84-87].

Four trials, two involving breast cancer patients [88,89], one in healthy subjects [61], and one in women undergoing breast biopsy or definitive surgery for breast cancer [90] were identified in which breast biopsies were taken before and after exposure to either isoflavone supplements or ISP (Table 1). In no case did the intervention lead to an increase in breast epithelial cell proliferation, which was used in these studies as a marker of potential tumor promotion. Daily isoflavone intake in these trials ranged from 36 [61,91] to >100 mg [88,89] and study duration from 2 weeks [90] to one year [89]. In comparison, postmenopausal ET results in modest variable increases in proliferation, while estrogen plus progestin therapy (EPT) results in more significant increases in breast cell proliferation [92,93].

**Table 1:** Clinical effects of isoflavones and soy protein on markers of breast cancer risk

Author, Year/(Reference)	Subject No./Intervention Product/Isoflavone Exposure (mg/d)^1^	Study Length	Subject Description	Sampling Method	Primary Measures of Interest	Results		
Breast Biopsies								

Cheng, 2007/(61)	25/placebo 26/tablets/36	12 wk	Healthy post-menopausal women, age range, 49–69 y; mean age, ~57	Middle-needle biopsy of breast tissue using ultrasound to identify glandular tissue	ERα, ERβ, ERβcx,^2 ^and PRα/β^3 ^expression, Ki67	NSE^5 ^for any measure. The proliferation marker, Ki67, was seen in 0% to 3% of samples, and no significant change was induced by isoflavone treatment.		
Sartippour, 2004/(88)	26/historical controls 17/tablets/120	~22 d	Women with invasive/infiltrating breast cancer diagnosed by core-needle biopsy; mean age, ~61 y	Breast cancer biopsies and surgical specimens	ER & PR expression, p53, her-2/neu, DNA flow analysis, apoptosis and mitosis	NSE but trend toward an ↑ in the ratio of cells undergoing apoptosis versus mitosis in isoflavone (IF) group		
							Apoptosis/Mitosis*	
							Control	Isoflavone
						Pre	6.5 ± 7.0	5.5 ± 4.7
						Post	3.3 ± 3.4	5.8 ± 8.3
						*Apoptosis and mitosis counts/high-power fields, means ± SD		
Palomares, 2004/(89)	9/placebo 9/tablets/100	11.7 mo	Postmenopausal women previously diagnosed with in-situ or early stage invasive (Stage I-II) breast cancer; mean age, 56.9 ± 1.4 y	Ultrasound-guided 14-gauge core biopsies of the contralateral breast	Histology, ER/PR expression, Ki67	NSE for any measure.		
						Breast tissue histology*	PBO	IF
						Normal	5	5
						Hyperplasia w/o atypia	2	2
						Hyperplasia with atypia	0	1
						Inadequate	2	1
						Ki67 index* (mean)	5.9%	5.4%
						(SD)	5.2%	6.5%
						* values represent number of subjects		
Hargreaves, 1999/(90)	53/UD^5^ 28/UD + 60 g soy protein/~45	14 d	Premenopausal women undergoing breast biopsy or definitive surgery for breast cancer;^6 ^mean age, ~33 y	Grossly normal breast tissue (~1 cm^3^) excised at least 1 cm from the site of the lesion.	ER/PR expression, thymidine and Bcl-2 labeling, Ki67	NSE for any measure		
							Ki67 labeling index	
							Wks 1 & 2	Wks 3 & 4
						Control	3.16 ± 3.08	6.03 ± 4.27
						Soy	4.76 ± 6.16	6.17 ± 7.0
						Values are mean ± SD		

Mammograms (Breast Tissue Density)								

Tice (in press)/(98)^7^	23/UD+25 g casein 24/UD+25 g ISP^8^/50	6 mo	Premenopausal women at high risk of breast cancer (defined by Gail risk ≥ 1.67% and mammographic breast density ≥ 50%)	Timed to late follicular phase (Day 10). Computer-aided contour method. Pre/post films read paired in random order at close of study, CC view and single reader		NSE		
Powles, 2008/(99)	Premenopausal 111/tablets/40^9^ 111/Placebo/0 Postmenopausal 8/tablets/40^9^ 11/placebo/0	3 y	Healthy women aged between 35 and 70 y with at least one first-degree relative with breast cancer	Mammograms were conducted on both breasts. All film images were digitalized and breast density was determined from the digital or digitalized images. Breast density was measured on a scale of 0–100 with higher figures representing more dense breasts		Mean change (%) from baseline plus 95% CI Premenopausal		
						Isoflavone	3.03	(-5.53 – -0.54)
						Placebo	6.60	(-9.04 – -4.16)
							Postmenopausa	
						Isoflavone	-6.9	(-11.6 – -2.1)
						Placebo	-8.0	(-15.7 – -0.2)
Maskarinec, 2004/(96)	103/UD 98/2 servings soyfoods/~50	~2.2 y	Healthy premenopausal women; average age, ~43 y	Computer-assisted density assessment. All mammograms for 1 woman were assessed during the same session, but the reader was unaware of the group status or the time sequence of the mammograms.		Breast tissue density (%)		
							Control	Soy
						Baseline	48.1 ± 25.2	45.6 ± 23.3
						Final	43.2 ± 24.3	40.5 ± 23.7
						Change	4.1 ± 10.2	2.8 ± 9.6
						Values are means ± SD. NSE		
Atkinson, 2004/(97)	61/Placebo 56/tablets/43.5^9^	12 mo	Postmenopausal women with Wolfe P2 or DY breast patterns; age range, 49–65 y; mean age, ~55	Percent densities assigned by drawing and measuring a cross on a 100 mm line (representing 0–100% density)		Reader 1: in the placebo and isoflavone groups respectively, 22% and 18% of women changed to a more lucent Wolfe pattern, 78% and 80% did not change, and 0% and 2% changed to a more dense Wolfe pattern. Reader 2: in the isoflavone and placebo groups, respectively, 15% and 19% of women changed to a more lucent Wolfe pattern, 84% and 80% did not change, and 1% and 1% changed to a more dense Wolfe pattern. NSE of isoflavone treatment		
Maskarinec, 2003/(95)	15/UD 15/UD + tablets/76	~12 mo	Healthy pre-menopausal women; mean age, 42 y	Computer-assisted density assessment. Left and right cranio-caudal views of the mammograms (all free of malignancies) were scanned into a PC using a Cobrascan CX-612-T digitizer.		Percent breast tissue density		
							Control	Soy
						Initial	49.5 ± 12.6	34.6 ± 18.8
						Final	49.9 ± 12.8	37.1 ± 16.5
						Values are means ± SD. NSE		

Nipple Aspirate Fluid (NAF)								

Qin, 2007/(103)	15/tablets/24 19/tablets/42	~1 mo	Premenopausal women with no history of atypia, *in situ *or invasive breast cancer; age range, 19–54; median, ~37 y	NAF was collected before and after one menstrual cycle. Samples from the left and right breast were kept separate	Estrogen marker, complement (C)3 and cell cytology	NSE		
Hargreaves, 1999/(90)	53/UD 28/UD + 60 g soy protein/~45	14 d	Premenopausal women undergoing breast biopsy or definitive surgery for breast cancer;^6 ^mean age, ~33 y	NAF obtained by bimanual, four-quadrant compression of the breast. Fluid was collected into capillary tubes, and the volume of neat nipple secretion was calculated by multiplying the length (in millimeters) of nipple fluid in the tube by the cross-sectional area of the capillary tube lumen	Apolipoprotein D (apoD) and pS2 levels	Statistically significant ↑ and ↓ in pS2 and apoD levels, respectively (*P *≤ 0.002).		
Petrakis, 1996/(102)	24/UD + 37.4 g ISP/75	6 mo	Premenpausal (n = 14) and postmenopausal women (N = 10)	NAF was obtained with a Sartorius-type breast pump consisting of a 15-cc syringe attached to a small cup by a short piece of plastic tubing	NAF volume, gross cystic disease fluid protein (GCDFP-15) concentration, and NAF cytology.	Statistically significant ↑ in fluid volume and ↓ in GCDFP-15 in premenopausal women only. Epithelial hyperplasia in 7 of 24 women during and after ISP intake.		

^1 ^Daily isoflavone intake expressed as aglycone units; ^2 ^ER, estrogen receptor; ^3 ^PR, progesterone receptor; ^4 ^NSE, no statistically significant effects; ^5 ^UD, usual diet; ^6^Women diagnosed with benign breast disease included fibroadenoma (n = 38), reduction mammoplasty (n = 10), fibrocystic masses (n = 9), duct ectasia (n = 6), sclerosing adenosis (n = 3), lipoma (n = 1), and accessory breast removal (n = 1); thirteen cases of breast cancer were of the invasive ductal type, and 3 were ductal carcinoma *in situ*; fourteen patients were confirmed as taking oral contraceptives at the time of surgery, and 61 were parous; twenty (71.4%) patients completed 13–14 days of soy supplementation, 4 (14.3%) completed 10–12 days, and 4 (14.3%) completed 8–9 days of soy supplementation; however, all patients said they had taken the last soy tablet 24 h before surgery; ^7 ^Details are described in reference; ^8 ^ISP, isolated soy protein; ^9 ^Isoflavones derived from red clover.

In one of the trials performed in healthy subjects, 28 postmenopausal women consumed 60 g textured vegetable (soy) protein containing 45 mg isoflavones for 2 weeks. No statistically significant effects on cell proliferation or several other estrogen-responsive markers were found, including progesterone receptor expression, Bcl-expression, and cells undergoing apoptosis and mitosis. However, levels of the estrogen-regulated protein pS2 significantly increased subsequent to soy consumption within breast nipple aspirate (NAF) [69]. The second trial was a 12-week Swedish study in which 51 healthy postmenopausal women took a daily placebo or a supplement that provided 36 mg/d isoflavones [61]. No statistically significant effects of isoflavone treatment were seen on cell proliferation or several other indicators of estrogenic effect (Table 1).

Two other pilot studies involving breast cancer patients also failed to find an effect of isoflavone supplements on breast cell proliferation. The intervention period averaged 23 days in one study [88] and a year in the other [89]. In both studies subjects were exposed to ≥ 100 mg isoflavones per day; however, the one-year study included only 9 women per group and is published only as an abstract. Interestingly, in this study, biopsies taken from the contralateral breast revealed an increase in breast cell proliferation at baseline, which supports the idea that the "healthy" contralateral breast of breast cancer patients may be at an increased risk of developing a tumor [94].

In addition to the lack of effect on cell proliferation, none of the five studies conducted (three in premenopausal [95-98], one in postmenopausal women [97] and one involving both pre- and postmenopausal women [99]) found that isoflavone exposure from soyfoods, ISP, or soybean- or red clover-derived supplements significantly affected breast tissue density (Table 1). Greater breast tissue density is associated with increased breast cancer risk and as was the case for cell proliferation, the lack of effects of isoflavones on breast tissue density generally contrasts with the effects of ET and EPT (see below) [100,101].

Two additional clinical trials are worthy of comment (Table 1). In one, breast NAF was collected for a total of one year [102]. Samples were taken over three months prior to soy exposure, then for 6 months during which women consumed 37.5 g ISP that provided 75 mg isoflavones daily, and then for 3 months after discontinuation of soy intake [102]. Hyperplastic epithelial cells were noted in 7 of 24 (29.2%) women (4 premenopausal and 3 postmenopausal) while consuming soy whereas prior to soy consumption hyperplastic cells were noted in only 1 of 24 women (4.2%) [102]. The authors concluded that these findings suggest that soybean isoflavones exert an estrogenic stimulus on breast tissue. However, it is important to point out that this was a pilot study with several limitations including the lack of a control group, a high dropout rate (only 15 of 37 subjects finished the 12-month regimen), and the fact that hyperplastic epithelial cells in the NAF persisted far beyond cessation of soy protein intake. Furthermore, a more recent study involving 34 premenopausal women found that isoflavones had no impact on breast cell cytology after one month exposure to either ~24 or 42 mg/d isoflavones [103]. While the available trials examining breast proliferation and density have found no statistically significant effects of isoflavone-containing products it is important to recognize that many of these studies involved small sample sizes or were relative short in duration.

Finally, two epidemiologic studies were identified that examined the relationship between soy or isoflavone intake and breast cancer survival. The first found that soyfood intake was unrelated to survival over the 5.2 year follow-up period [104]. In this study, approximately 63% of the 1001 Chinese breast cancer cases (out of 1459 subjects in the total cohort) for whom data on receptor status was available were ER+. In the other study, when comparing the fifth versus the first intake quintiles, isoflavone intake was associated with a reduced risk of all-cause mortality over the approximate 5-year follow-up period [105]. Isoflavone intake was also associated with a marginal reduction in risk of breast cancer-specific mortality, although the effect was not statistically significant. Of note, the isoflavone intake cutoffs for the fifth quintile were only 7.48 and 0.60 mg/d for all-cause and breast cancer-specific mortality, respectively, and the percentage of ER+ patients among the 1210 subjects was not indicated.

### Estrogen and breast cancer risk

Since the estrogen-like effects of isoflavones are at the core of the soy-breast cancer controversy, understanding the relationship between estrogen and breast cancer provides a potentially useful perspective. There is a large amount of evidence that endogenous estrogens are involved in the etiology of certain types of breast cancer [106,107]. Endogenous estrogens increase breast epithelial proliferation and may facilitate growth of estrogen-sensitive neoplastic or preneoplastic cells [108,109]. Many of the major epidemiologic risk factors for breast cancer also relate to endogenous estrogen exposure. For example, greater lifelong exposure to ovarian estrogen – as occurs with early menarche and late menopause – is associated with increased breast cancer risk [110-112], whereas oophorectomy reduces risk in premenopausal women [113-115]. In postmenopausal women, higher endogenous circulating concentrations of estrogen [116,117] are associated with increased risk, as are obesity and alcohol intake, both of which result in higher endogenous estrogen levels [112,118]. Conversely, treatment with tamoxifen and raloxifene, which inhibits ER activity in the breast, and aromatase inhibitors, which reduce endogenous estrogen production, are effective for treating and preventing ER+ breast cancer [119,120].

The risk of breast cancer associated with exogenous estrogen exposure is less clear, however, due in part to recent results of the Women's Health Initiative (WHI). This study consisted of two large parallel randomized, double-blind, placebo-controlled clinical trials of hormone therapy designed to evaluate effects of conjugated equine estrogens (CEE) alone (for women with prior hysterectomy) or in combination with the progestin medroxyprogesterone acetate (MPA). In the WHI Estrogen + Progestin Trial, use of CEE + MPA led to a 26% increase in breast cancer risk (38 vs 30 cases per 10,000 person-years) which was highly significant in the weighted analysis (P < 0.001) [121]. However, in the WHI Estrogen-Alone Trial, after an average of 7.1 years of follow-up, women assigned to CEE alone at 0.625 mg/d were 18% *less *likely to develop invasive breast cancer compared to women in the placebo group (26 vs 33 cases per 10,000 person-years; P = 0.09) [122]. When the latter analysis was restricted to adherent subjects, risk in the CEE group was reduced by one-third (P = 0.03), while the incidence of localized breast carcinoma and ductal carcinoma were lower by 31% and 29%, respectively [123].

The reason for the marginal reduction in breast cancer risk associated with estrogen-alone therapy in the WHI trial is currently unknown. Prior epidemiologic evidence regarding ET effects on breast cancer risk is mixed but generally indicates either no significant effect or a modest increase in risk with long-term exposure [124-128]. Variation within and across observational studies may relate to a variety of factors, including subject selection, screening frequency, duration of hormone use, hormone formulations and doses, and patient characteristics such as reproductive history, body mass index, and background endogenous estrogen context. Nevertheless, overall risks from observational studies are generally small for ET and notably lower than those reported for combined EPT, consistent with WHI results. Importantly, studies of ET use in breast cancer survivors (generally for periods < 5–10 years) also indicate minimal if any risk for recurrence or mortality [129-135].

Direct effects of ET (CEE in particular) on breast proliferation and density are generally modest and less than those seen with EPT. In one of the few clinical studies to assess breast proliferation following ET and EPT, postmenopausal women taking EPT but not ET had significantly greater breast epithelial Ki67 expression in terminal ductal lobular areas [82]. In this study, ET was associated with modestly higher percent breast epithelial area (~15%) compared to the control group (~7%; P = 0.01), while EPT resulted in greater density beyond that seen with ET (~24%; P = 0.02 compared to ET).

Consistent with these findings, the Postmenopausal Estrogen/Progestin Interventions (PEPI) randomized placebo-controlled clinical trial reported a non-significant change in mammographic density of +1.2% after 1 year of CEE treatment compared to significant increases of +3.1 to +4.8% for different EPT regimens [136]. In the WHI, absolute changes in mammographic density were not reported, although CEE resulted in a greater overall percentage of women with abnormal mammograms (36.2% for CEE compared to 28.1% for placebo) [123].

In conclusion, while there is general agreement that endogenous estrogen exposure has an important role in the etiology of breast cancer, the extent to which postmenopausal exogenous estrogen exposure affects risk is much less certain. Current evidence suggests that use of oral ET (particularly CEE) by relatively healthy postmenopausal women for periods < 10 years has very low if any risk for breast cancer and minimal to no effect on breast cancer recurrence or mortality in breast cancer survivors. This information provides a sensible context for considering the potential adverse effects on dietary soyfoods or isoflavones. Given the low overall risk associated with pharmacologic estrogen exposure, how reasonable is it to expect that any weak estrogen-like effects of soy-derived isoflavones (which have yet to be clearly demonstrated in the breast) may increase breast cancer risk or worsen the prognosis of breast cancer patients?

## Summary and conclusion

Isoflavones are phytoestrogens which interact with ERs and generally function as weak estrogens in rodent and cell culture models. These estrogen-like effects have raised concern regarding soy/isoflavone consumption, particularly in the case of postmenopausal women at high risk for breast cancer. Currently there is little evidence to suggest that any potential weak estrogenic effects of dietary isoflavones have a clinically relevant impact on breast tissue in healthy women. Limited data suggest this is also the case for breast cancer survivors. This evidence includes multiple trials showing no effects on breast proliferation or mammographic density and considerable epidemiologic data showing either no effect or a modest protective role of soy/isoflavone intake on breast cancer risk. Tangential support for this idea is also provided by recent clinical trial findings regarding exogenous ET (in the form of CEE) showing a marginal decrease in risk of invasive breast cancer. Based on this evidence it seems unlikely that isoflavone consumption at dietary levels (i.e. <100 mg/day) elicits adverse breast cancer-promoting effects in healthy women or breast cancer survivors not undergoing active treatment. Findings from one rodent study showed that genistein may interfere with concurrent tamoxifen treatment, suggesting that breast cancer patients taking a SERM may need to limit soyfood intake and avoid isoflavone supplements. Currently there are no data to support the idea that soyfoods or isoflavone supplements improve the prognosis of breast cancer patients. Available data for ET effects on breast cancer recurrence and mortality provide some assurance for breast cancer patients that soyfoods/isoflavone supplements, when taken at dietary levels, do not contribute to recurrence rates although more data are clearly needed to better address this issue.

## Abbreviations

CEE: conjugated equine estrogens; E2: 17β-estradiol; ER: estrogen receptor; ER+: estrogen receptor positive; ET: estrogen therapy; EPT: estrogen plus progestin therapy; ISP: isolated soy protein; MPA: medroxyprogesterone acetate; NAF: nipple aspirate fluid; NSE: no statistically significant effect; SERM: selective estrogen receptor modulator; WHI: Women's Health Initiative.

## Competing interests

M.M. is president of Nutrition Matters, Inc., a nutrition consulting company with clients involved in the manufacture and/or sale of soyfoods and isoflavone supplements.

## Authors' contributions

MM and CEW were equally involved in the writing of this manuscript.
